# CD8‐positive peripheral T cell lymphoma in a patient following long‐term nivolumab for advanced lung adenocarcinoma: A case report

**DOI:** 10.1111/1759-7714.13966

**Published:** 2021-05-03

**Authors:** Keigo Koda, Mikio Toyoshima, Shusuke Yazawa, Atsuki Fukada, Haruhiko Sugimura, Takafumi Suda

**Affiliations:** ^1^ Department of Respiratory Medicine Hamamatsu Rosai Hospital Hamamatsu Japan; ^2^ Department of Tumor Pathology Hamamatsu University School of Medicine Hamamatsu Japan; ^3^ Second Department of Internal Medicine Hamamatsu University School of Medicine Hamamatsu Japan

**Keywords:** immune checkpoint inhibitor, nivolumab, lung cancer, T cell lymphoma

## Abstract

A 54‐year‐old male smoker presented with hemoptysis. Advanced lung adenocarcinoma, cT1cN3M1b, stage IVB, was diagnosed. Enlargement of multiple intraperitoneal and inguinal lymph nodes and peripheral atypical lymphocytosis appeared after 33 cycles of second‐line treatment with nivolumab, and a specimen obtained by left inguinal lymph node biopsy showed peripheral T cell lymphoma (PTCL), not otherwise specified. Lymphoma cells expressed CD3+, CD8+, and CD56+, but not CD4+ or PD‐1. Despite systemic chemotherapy with cyclophosphamide, hydroxydaunorubicin, oncovin, and prednisolone, the patient died of PTCL 864 days after the initial visit. The possible relationship between treatment with immune checkpoint inhibitors (ICIs) and PTCL development is discussed here.

## Graphical abstract

CD8‐postive peripheral T cell lymphoma developed in a patient with advanced lung adenocarcinoma after 33 cycles of second‐line treatment with nivolumab,The possible relationship between treatment with immune check inhibitors and T cell lymphoma development is discussed.

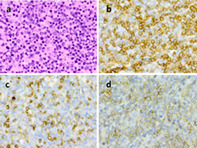

## INTRODUCTION

Immunotherapy with immune checkpoint inhibitors (ICIs) has become a powerful clinical strategy in the treatment of various malignant tumors.^1^ On the other hand, immune‐related adverse events (irAEs) are well known as major and problematic side effects of ICIs.^2^ However, information on serious side effects other than irAEs caused by ICI remains limited. We describe herein a case of CD8‐positive peripheral T cell lymphoma (CD8+ PTCL) after long‐term PD‐1 blockade by nivolumab for advanced lung adenocarcinoma, and discuss the possibility of a relationship between ICI treatment and development of CD8+ PTCL.

## CASE REPORT

**FIGURE 1:**
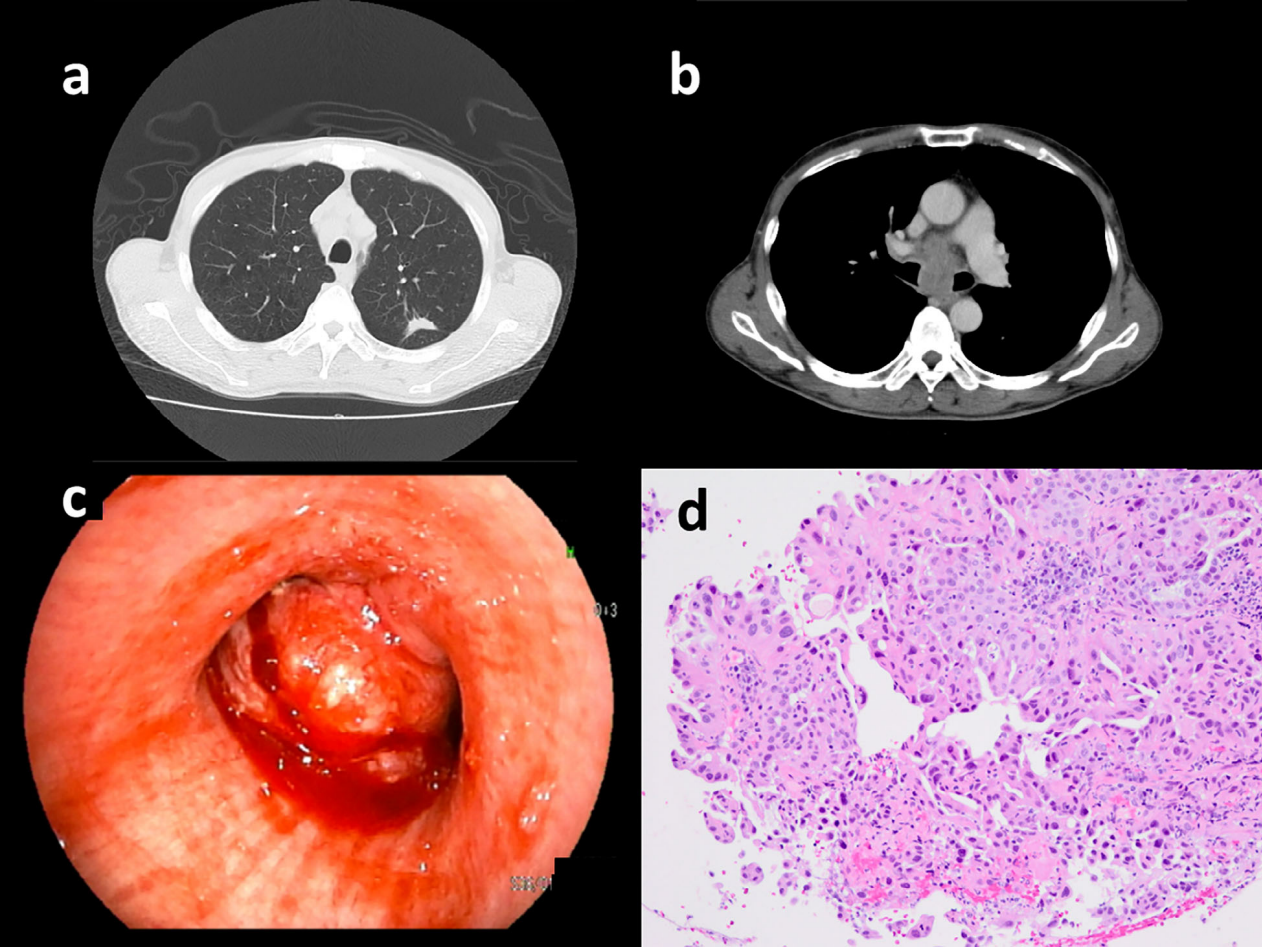
(a) Computed tomography (CT) of the chest shows a nodule 2.3 × 1.0 cm in size in the left upper lobe, and (b) enlargement of the mediastinal lymph nodes. (c) Bronchoscopic examination shows an intratracheal tumor, probably originating from a subcarinal lymph node metastasis. (d) A biopsy specimen obtained from the intratracheal tumor shows adenocarcinoma (hematoxylin and eosin stain, ×200)

**FIGURE 2:**
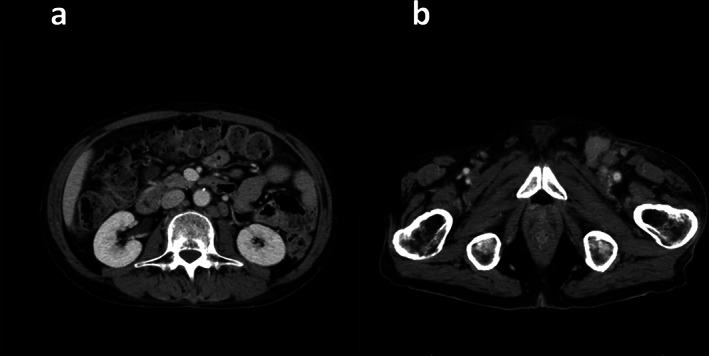
Computed tomography of the abdomen shows enlargement of multiple intraperitoneal lymph nodes, such as the para‐aortic lymph nodes (a) and inguinal lymph nodes (b)

**FIGURE 3:**
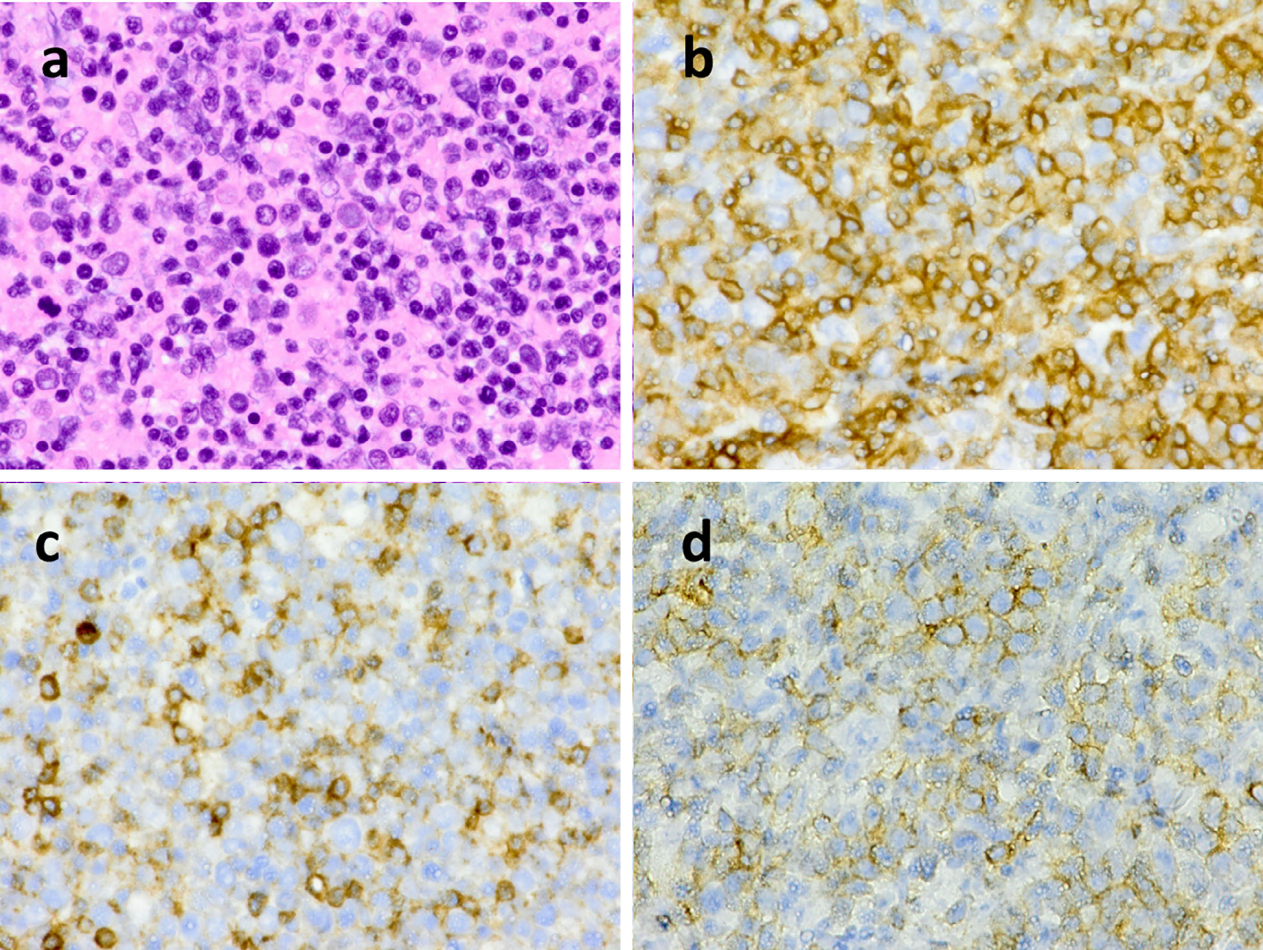
A biopsy specimen obtained from the left inguinal lymph nodes shows infiltration of atypical lymphocytes (a, hematoxylin and eosin stain, ×400) that are immunohistochemically positive for CD3 (b, ×400), CD8 (c, ×400), and CD56 (d, ×400)

**FIGURE 4:**
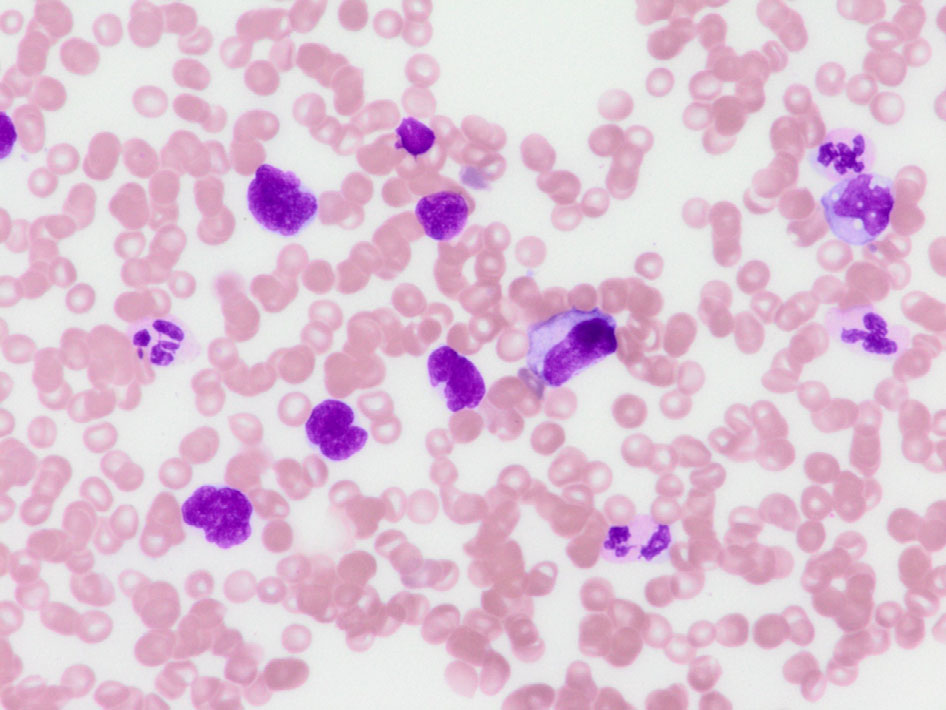
Smears of bone marrow aspirate obtained from the left posterior iliac crest show numerous flower cells (May‐Giemsa stain, ×1000)

**FIGURE 5:**
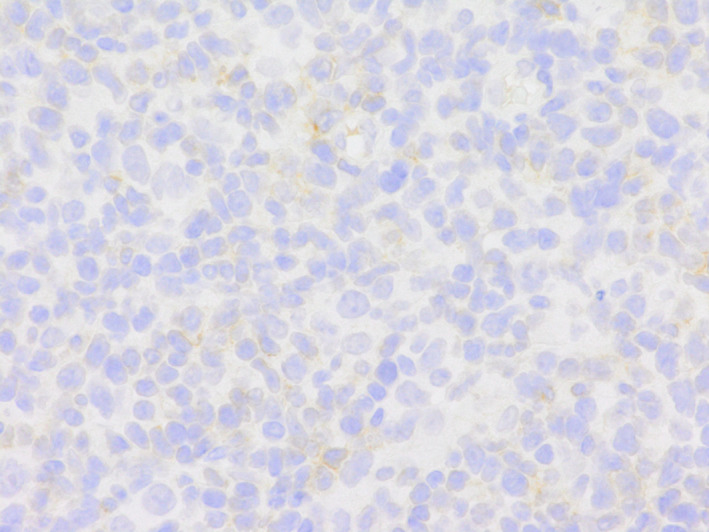
Lymphoma cells were immunohistochemically negative for PD‐1 (×400)

A 54‐year‐old man with a four‐week history of hemoptysis was referred to our hospital for detailed examination. His medical history was unremarkable, and he was a current smoker with a history of 25 pack‐years. Physical examination revealed no abnormalities. Laboratory findings showed elevated levels of serum carcinoembryonic antigen (50.5 ng/ml) and plasma D‐dimer (10.40 μg/ml). Computed tomography of the chest showed a nodule, 2.3 × 1.0 cm in size, in the left upper lobe, along with mediastinal lymphadenopathy (Figure 1(a), (b)). Bronchoscopic examination showed an intratracheal tumor, which was considered likely to have originated from a subcarinal lymph node metastasis (Figure 1(c)). A biopsy specimen obtained from the intratracheal tumor showed adenocarcinoma (Figure 1(d)). Neither gene mutation of epithelial growth factor receptor nor gene fusion of anaplastic lymphoma kinase were detected. The tumor positive score for PD‐L1 was 40%–49%. Diffusion‐weighted imaging of the brain revealed multiple high‐intensity micronodules, some of which were enhanced on T1‐weighted imaging with gadolinium contrast agent, suggesting multiple brain metastases and infarctions. The diagnosis was lung adenocarcinoma associated with Trousseau syndrome, cT1cN3M1b, stage IVb. Volume reduction for intratracheal tumor by laser ablation and placement of a Dumon Y‐stent was performed using a rigid bronchoscope. Shortly after these procedures, warfarin was prescribed for Trousseau syndrome. However, Broca aphasia and right hemiparesis appeared 10 days later. Warfarin was then switched to intravenous heparin, and whole‐brain irradiation (30 Gy in 20 fractions) was performed. Intravenous heparin was again switched to warfarin. After whole‐brain irradiation, four cycles of systemic chemotherapy with cisplatin and pemetrexed were administered. Neurological symptoms improved and mediastinal lymph node enlargement showed good partial response. Maintenance therapy was started with pemetrexed alone, but progression of mediastinal lymph node enlargement was seen after three cycles of maintenance therapy with pemetrexed. Nivolumab (240 mg every two weeks) was started as second‐line therapy. After three courses of nivolumab treatment, the patient started to complain of diarrhea, and immune‐related colitis (grade 2) was diagnosed on colonoscopic examination. Colitis resolved with the initiation of systemic prednisolone treatment (25 mg**/**day), which was tapered to chronic low‐dose prednisolone (5 mg**/**day). Because enlargement of the mediastinal lymph nodes regressed, treatment with nivolumab was continued. However, after 33 cycles of nivolumab treatment, severe colitis (grade 3) developed, so prednisolone was increased to 40 mg/day and nivolumab was discontinued. Colitis began to resolve, and the patient became asymptomatic. However, he noticed enlargement of the left inguinal lymph nodes, and laboratory findings showed increased peripheral atypical lymphocytes (2500/mm^3^) and elevated serum levels of lactate dehydrogenase (554 U/L). Negative results were obtained for serum anti‐human T‐lymphotropic virus 1 antibodies and anti‐human immunodeficiency virus antibodies. Computed tomography of the abdomen showed enlargement of multiple intraperitoneal and inguinal lymph nodes (Figure 2(a), (b)). A biopsy specimen obtained from the left inguinal lymph nodes showed infiltration of atypical lymphocytes that were immunohistochemically positive for CD3, CD8, and CD56 (Figure 3) and negative for CD4, CD20, and Epstein–Barr virus‐encoded small RNA (in situ hybridization). Smears of bone marrow aspirate obtained from the left posterior iliac crest showed numerous flower cells (Figure 4). CD8+ PTCL, not otherwise specified, stage IV, was thus diagnosed. The patient received four cycles of systemic chemotherapy with cyclophosphamide, hydroxydaunorubicin, oncovin, and prednisolone in the Department of Hematology. However, CD8+ PTCL had progressed, and his general condition worsened. Eventually, the patient died of CD8+ PTCL, 864 days after the initial visit to our hospital. No autopsy was performed. To investigate the mechanisms underlying the development of PTCL following nivolumab, we performed immunohistochemical stanning for PD‐1 in biopsy specimens obtained from the left inguinal lymph node using anti‐PD‐1 antibodies (ab52587; Abcam). PTCL cells were negative for PD‐1 (Figure 5).

## DISCUSSION

In this case, CD8+ PTCL developed with long‐term treatment (33 cycles) using the PD‐1 blocker nivolumab. The predominant immunophenotype in PTCL is CD3+ CD4+, and PTCLs expressing CD8+ CD56+ are relatively rare.^3^

A review of the English language literature found only two cases of T cell lymphoma related to ICI treatment. Zheng et al. reported a case of cutaneous CD56+ T cell lymphoma developing during pembrolizumab treatment for metastatic melanoma.^4^ In that case, T cell lymphoma developed after 20 months of treatment using pembrolizumab, and lymphoma cells expressed CD4+ CD8+, with a slight predominance of CD8+ cells. On the other hand, Anand et al. reported a case of T cell lymphoma developing after four cycles of pembrolizumab treatment for metastatic pleural tumor of unknown epithelial origin.^5^ In that case, lymphoma cells were immunohistochemically positive for CD3, but the results of immunohistochemical staining for CD4, CD8, and CD56 were not described. However, the authors identified a new *TET2* mutation in the clone representing the lymphoma by targeted exome sequencing. Based on the reported tumor suppressor function of PD‐1 in murine models,^6^ Anand et al. hypothesized that use of the PD‐1 inhibitor caused clonal proliferation of abnormal T cell clones leading to T cell lymphoma.^5^ Similarly, we speculate that the PD‐1 deletion demonstrated immunohistochemically as a second‐hit in CD8+ T cells with *TET2* mutation might have resulted in uncontrolled expansion of T cells, although we did not perform targeted exome sequence of the *TET2* mutation in this case.

In conclusion, here we present the first report of CD8 + PTCL developing after long‐term nivolumab treatment for advanced lung adenocarcinoma. Further investigations are needed to clarify the relationship between ICI treatment and development of T cell lymphoma.

## CONFLICT OF INTEREST

The authors have declared no conflicts of interest.
